# Can Videofluoroscopic Swallowing Kinematic Analysis Predict Recovery of Oral Intake in Postoperative Oral Cancer Patients Requiring Nasogastric Tube Feeding?

**DOI:** 10.3390/ijerph182212045

**Published:** 2021-11-16

**Authors:** Takuma Okumura, Koji Hara, Ayako Nakane, Chizuru Namiki, Kazuharu Nakagawa, Kohei Yamaguchi, Kanako Yoshimi, Mizue Toyoshima, Yoshiyuki Sasaki, Haruka Tohara

**Affiliations:** 1 Department of Dysphagia Rehabilitation, Division of Gerontology and Gerodontology, Graduate School of Medical and Dental Sciences, Tokyo Medical and Dental University, Bunkyo Ward, Tokyo 113-8549, Japan; tpegbbtdkt@gmail.com (T.O.); a.nakane.swal@tmd.ac.jp (A.N.); chizuru.n1025@gmail.com (C.N.); nakagerd@tmd.ac.jp (K.N.); yanma627@yahoo.co.jp (K.Y.); k.yoshimi.gerd@tmd.ac.jp (K.Y.); harukatohara@hotmail.com (H.T.); 2 Department of Critical Care Medicine and Dentistry, Division of Medically Compromised Geriatric Dentistry, Graduate School of Dentistry, Kanagawa Dental University, Yokosuka 238-8580, Japan; 3 Section of Nutrition Management, Tokyo Medical and Dental University Dental Hospital, Bunkyo Ward, Tokyo 113-8549, Japan; y.mizue.adm@cmn.tmd.ac.jp; 4 Department of Maxillofacial Surgery, Graduate School of Medical and Dental Sciences, Tokyo Medical and Dental University, Bunkyo Ward, Tokyo 113-8549, Japan; sasaki.prev@tmd.ac.jp

**Keywords:** oral cancer, nasogastric tube, oral intake, swallowing kinematics, videofluoroscopic swallowing

## Abstract

This retrospective study determined the significant predictive factors for the number of days required to remove nasogastric tubes (NGTs) after surgery in patients with oral cancer (OC). In this study, patients underwent a videofluoroscopic swallowing study (VFSS) approximately 2 weeks after surgery. Videofluoroscopic images were analyzed, and variables such as swallowing and swallowing kinematics were measured. Patient characteristics, swallowing kinematics, and swallowing results were assessed using a Cox proportional hazards model. This study assessed 129 participants (66 men, 63 women, mean age: 69.0 ± 14.1 years) with nine types of cancer. The Cox proportional hazard ratio revealed that sex, body mass index before surgery, radiotherapy and/or chemotherapy, dysphagia before surgery, normalized pharyngeal constriction ratio, upper esophageal sphincter (UES) opening, and laryngeal vestibule disclosure (LVC) disorder were predictive factors for the removal of NGTs when adjusted for age. The study identified several predictive factors for the removal of NGTs and oral intake recovery in patients with OC. Regarding swallowing kinematics, UES opening is the most significant predictive factor. After surgery for OC, VFSS should be performed to assess safe eating methods and predict the recovery of oral intake and removal of the NGT.

## 1. Introduction

Dysphagia is common in postoperative patients with oral cancer (OC) [1]. Surgery and chemoradiation may affect the kinematics of the oropharyngeal structures during swallowing, which often contributes to dysphagia [2,3]. A nasogastric tube (NGT) is often used in clinical settings to provide adequate nutrition to patients with dysphagia. However, it has been reported that long-term use of NGTs is associated with complications such as nasal wing lesions, chronic sinusitis, gastroesophageal reflux, and poor performance in activities of daily living [4]. It has also been reported that prolonged NGT use adversely affects swallowing function due to disuse [5]. In addition, it may affect a patient’s mental status, as an association between dysphagia and mental status has been reported [6,7]. Therefore, early removal of the NGT should be considered in the clinical setting. In addition, clarifying the factors related to the early removal of NGTs in postoperative patients with OC is important.

It has been reported that post-surgery swallowing function recovery is better in patients with a higher functional oral intake scale (FOIS) score pre-surgery, smaller tumors, and no requirement for radiotherapy [8]. In addition, the administration of chemotherapy and radiotherapy to patients with head and neck cancer (HNC) can prolong the time to removal of the NGT [9]. Considering the adverse effects of surgery for OC on oral and pharyngeal structures, the relationship between prolonged time until removal of the NGT and postoperative swallowing function should be elucidated. However, to date, no study has investigated this issue. Moreover, there are fewer reports on the most effective strategies to manage dysphagia after OC surgery than those on strategies to manage dysphagia caused by common diseases such as stroke or neuromuscular diseases [3].

Videofluoroscopic swallowing study (VFSS) is widely used as the gold standard for evaluating swallowing function, including the presence of aspiration and swallowing kinematics [10,11]. Although it has been reported that aspiration in postoperative dysphagia associated with OC is related to videofluoroscopic (VF) kinematic swallowing analysis features such as hyoid movements [12] and pharyngeal constriction [13], there has never been a report on the relationship between removal of the NGT and swallowing function assessed by VFSS. The present study aimed to investigate whether the results of VFSS, including the presence of aspiration and swallowing kinematics’ parameters, can predict the removal of an NGT in postoperative OC patients with tube feeding.

## 2. Materials and Methods

### 2.1. Research Design and Participants

A retrospective cohort study of 213 patients treated for OC between March 2015 and September 2021 at the Department of Oral Surgery of Tokyo Medical and Dental University Dental Hospital was conducted. The patients were referred to the Department of Dysphagia Rehabilitation of Tokyo Medical and Dental University Dental Hospital for swallowing assessment after surgery. All participants in this study underwent VFSS and a fiberoptic endoscopic evaluation of swallowing (FEES) approximately 2 weeks after surgery. Based on the results of VFSS, the participants were provided with an appropriate diet (regular, easy to chew, soft and bite-sized, minced, moist, pureed, or liquidized). If required, they were also recommended compensatory strategies during meals such as maintaining a reclined posture, head and neck flexion, or head rotation and/or swallowing maneuvers such as voluntary cough after swallowing or alternate swallowing to avoid aspiration or pharyngeal residue. All participants underwent daily dysphagia rehabilitation with dentists or nurses after VFSS.

The exclusion criteria were as follows: defocused VF image, no attempt to swallow 2% thick 4 cc during VF (which was used in the analysis of swallowing kinematics), missing data, neuromuscular disease, or stroke. This research was approved by the Dental Research Ethics Committee of Tokyo Medical and Dental University (D2015-636). Written informed consent was obtained from all participants.

The researcher collected data related to the patients’ medical history and records, including the history of surgery for OC, sex, age, body mass index (BMI) before surgery, the use of chemotherapy and/or radiotherapy before and after surgery (CR), and the presence of dysphagia before surgery. The presence of dysphagia before surgery was assessed using FOIS [14], where a score of <6 was defined as the presence of dysphagia. Underweight was defined as a BMI of <18.5 before surgery. Individuals aged > 65 years were considered elderly.

### 2.2. VF Procedure and Analysis

VF examination was performed for the first time in patients approximately 2 weeks after surgery. The examiner instructed the participant to swallow 4 cc liquid of a predetermined viscosity (2% thick, 1% thick, 0.5% thick, and thin liquid, in that order). Each viscosity trial was conducted once or twice, as needed, to assess the swallowing function of participants precisely. Then, if the participant aspirated, the examination was completed. In short, these participants did not drink a thinner liquid than those who had aspiration.

Lateral VF images were taken at a speed of 30 frames/s using the digital X-ray television system Flexavision (Shimadz Medical Systems Corp, Osaka, Japan). Video was recorded with a digital recorder (DV-AC82; Sharp Corporation, Osaka, Japan). The VF video was subsequently converted into VF images, which were analyzed according to a previous study as follows: Each swallow was assessed to determine penetration aspiration scale (PAS). A 2% 4 cc trial lateral image was used for the other VFSS analysis. This analysis was based on pixel-based measurements using Image J software and was normalized by the length of the anterior inferior corner of the C2-C4 vertebrae.

#### 2.2.1. Aspiration 

The presence of aspiration in each swallow was assessed using the PAS [15]. A PAS score of >5 was defined as the presence of aspiration. Among the trials, the worst PAS score was used in the analysis.

#### 2.2.2. Swallowing Kinematics

Laryngeal vestibule closure (LVC) [16] was assessed in a previous study with two ratings: complete or incomplete. Incomplete refers to cases of partial closure, including space or bolus in the laryngeal vestibule during and after swallowing. A rating of complete was considered good, whereas a rating of incomplete was considered poor. The width of the upper esophageal sphincter (UES) opening was measured as the narrowest point between cervical vertebrae 3 and 6, measured at the point of maximum distension during the swallow, as described in a previous study [17]. Oral phase dysphagia (OPD) was determined at our own discretion via VFSS observation as participants who were unable or took a long time to transport a bolus from the oral cavity to the pharynx or had a large amount of residue in the oral cavity after swallowing. Hyoid bone displacement was measured as follows: the distance between the resting and maximal anterior hyoid positions, as described in a previous study [18]. These were measured based on the *X*–*Y* axis, the line joining points of the anterior inferior corner of C2 and C4 as the *Y*-axis, and appendicular to the *Y*-axis as the *X*-axis. The normalized pharyngeal constriction ratio (PCR_N_) was measured using the smallest amount of unobliterated air space and bolus visible in the pharynx. In addition, the largest amount of unobliterated air space in the pharynx after swallowing in the resting position was measured, and PCR_N_ was accordingly calculated, as described in a previous study [19].

### 2.3. Intraclass Correlation Coefficients (ICC)

We calculated the intra-rater and inter-rater ICC of aspiration, LVC, OPD, hyoid bone movements, UES opening, PCR_N_, and nasopharyngeal closure in 30% of all randomly selected participants. The inter-rater ICC for the variables was in the range of 0.743–0.99, whereas the intra-rater ICC was in the range of 0.833–0.952. These measurements were highly reliable.

### 2.4. Main Outcome and Study Analysis

The main outcome measure was removal of the NGT. When the patient received an adequate amount of nutrition per day via oral intake, the NGT was removed. An adequate amount of calories was calculated based on the Harris–Benedict equation [20]. The number of days from surgery to removal of the NGT was defined as the number of days required to remove the NGT. These data were collected from medical databases.

The Shapiro–Wilk test was used to determine normal or non-normal distributions of continuous data. Continuous data, which were determined to be non-parametric, were dichotomized based on the median. Cox proportional hazards models were used to determine the factors that can predict removal of the NGT after adjusting for age, sex, BMI before surgery, dysphagia before surgery, chemotherapy and radiation before/after surgery, aspiration, and swallowing kinematics. To construct an appropriate model, we included potential confounding variables and clinically significant variables whose importance in swallowing kinematics has been reported previously [16,17,18,19]. These variables could be measured quantitatively and are associated with dysphagia after surgery. Clinically, reasonable combinations were applied. In the Cox proportional hazards models, we set removal of the NGT as the event, with percutaneous endoscopic gastrostomy (PEG) and transfer, aspiration pneumonia, and wound problems as censors. Statistical significance was set at *p* < 0.05. All statistical analyses were performed using EZR software, version 1.54 (Saitama Medical Center, Jichi Medical University, Saitama, Japan), a graphical user interface for R (The R Foundation for Statistical Computing, Vienna, Austria) [21].

## 3. Results

A total of 198 participants were included in the study. Patients were excluded from the study for the following reasons: 51 because of missing data, 10 because of postoperative wound problems, and 8 because of neuromuscular disease. Finally, 129 patients (66 men, 63 women; mean age: 69.0 ± 14.1 years) were analyzed in this study. Table 1 shows the demographic features of the patients. Before surgery, 84.5% of the participants had regular meals and thin liquids without any swallowing problems. A total of 109 patients (84.5%) underwent a tracheotomy during surgery. No patients had a tracheal cannula when VFSS was performed for the first time. After surgery, 16 patients (12.4%) could not undergo removal of the NGT, which was used as a censor in the Cox proportional hazards model. Among them, 11 (8.5%) patients underwent PEG, 3 patients had aspiration pneumonia (2.3%), 1 patient was transferred to the hospital for acute renal failure (0.8%), and one patient had a wound infection (0.8%) after surgery before removal of the NGT. The FOIS scores after surgery were at level 1 (nothing by mouth) in 9 patients (7.0%), level 2 (tube dependency with minimal attempts at food or liquid intake) in 4 (3.1%), level 3 (tube dependency with consistent oral intake of food or liquid) in 47 (36.4%), level 4 (total oral diet of a single consistency) in 67 (51.9%), and level 5 (total oral diet of multiple consistencies, requiring special preparation or compensations) in 2 (1.6%). Table 2 presents the period until removal of the NGT according to the type of cancer. The median (IQR) period until removal of the NGT in the overall cohort was 20.5 (15.8–28.0) days, with no difference according to the cancer type. Table 3 summarizes the results of risk factor analysis using the Cox proportional hazards model. The results showed that sex, BMI before surgery, dysphagia before surgery, CR, LVC, PCRN, and UES opening affected the period until removal of the NGT when adjusted for age group.

Our results showed that PCR_N_, UES opening and LVC, CR, BMI before surgery, sex, and dysphagia before surgery were predictors of removal of the NGT in patients with OC requiring tube feeding.

## 4. Discussion

### 4.1. Significant Variables for Removal of the NGT

Regarding PCR_N_, it is important for swallowing to create pressure in the pharynx when transporting a food bolus from the pharynx to the esophagus. During this motion, positive pressure in the pharynx is created by the pharyngeal constricting muscle, nasopharyngeal closure, and tongue base contacting the pharyngeal wall [22]. Damage by surgery and CR to the oropharyngeal structures in patients with OC causes insufficient pharyngeal constriction, resulting in a large PCR_N_. A larger PCR_N_ reportedly causes postoperative dysphagia after surgery for HNC [23]. These findings suggest that PCR_N_ is significantly associated with removal of NGTs.

UES opening creates negative pressure during swallowing with cricopharyngeal muscle relaxation and thyroid cartilage elevation [22]. Manometric studies have shown that food transportation from the pharynx to the esophagus requires the negative pressure created by UES opening and the positive pressure created by pharyngeal constriction [24]. In this study, the impaired UES opening with a larger PCR_N_ was associated with a higher risk of prolonging the time until removal of the NGT. Therefore, our results were consistent with those of the previous study [22,24].

A previous study evaluated the relationship between dysphagia after surgery and the degree of UES opening during swallowing via VFSS in patients with HNC [25]. They found that impaired UES opening due to surgical invasion of the oropharyngeal structure resulted in poor oral intake recovery and a prolonged period until removal of the NGT. As mentioned above, UES is achieved by cricopharyngeal muscle relaxation and thyroid cartilage elevation. However, in this study, none of the participants had cricopharyngeal muscle relaxation impairment before surgery. Therefore, thyroid cartilage elevation might have been an important factor associated with removal of the NGT in the present study.

Our results showed a relationship between LVC and the days until removal of the NGT. Laryngeal closure is composed of many movements around the larynx, such as thyroid cartilage elevation, true vocal cord closure, arytenoid cartilage adduction, and epiglottis inversion [26]. In the present study, we assessed laryngeal closure only to measure the space in the larynx in the swallowing reflex or the presence of penetration, based on a previous study [16]. In addition, true vocal cord closure and arytenoid cartilage adduction can only be assessed via endoscopy. Epiglottic tilting during swallowing was not evaluated in the present study, which may have affected the prevision of laryngeal closure evaluation. However, the hazard ratio of the group with good LVC function was approximately twice as good as that of the group with poor LVC function. Thus, measuring LVC via VFSS might reflect laryngeal closure capacity, which is essential for safe swallowing.

CR-caused dysphagia has been frequently documented [2,3]. In addition to the other aforementioned risk factors, CR can cause mucositis, pain, nausea, dysgeusia, and impaired swallowing kinematics [27]. It is very common for patients who have received CR to require more time to remove the NGT and recover oral intake than those who do not receive CR.

In this study, BMI was a significant variable according to the Cox proportional hazard analysis. In general, a high BMI is preferable in patients treated for OC to avoid treatment delays and prolonged hospitalization [28,29]. In contrast, patients with a higher BMI had a more prolonged time until removal of the NGT than those with a smaller BMI in this study. The reason for this remains unclear. However, there are three possible explanations. First, in this study, the median BMI was 22.1 (IQR: 20.0–24.2). Only 14 patients (10.9%) had a BMI < 18.5 and two (1.6%) had a BMI < 16.5. Thus, the small number of patients with lower BMIs might have influenced the findings. The second explanation is that the relationship between BMI and the amount of calories and meals needed by patients might be related to removal of the NGT. In other words, patients with a higher BMI need to consume larger amounts of calories than those with a lower BMI. In this study, the appropriate amount of calories via the mouth needed by patients after surgery to remove the NGT was estimated using the Harris–Benedict equation [20]. In addition, due to dysphagia after surgery, resection, or CR, patients could eat only shapeless diet forms, such as minced and moist, pureed, and liquidized, after starting eating, or they required direct training for dysphagia. These diet forms contain a larger amount of water after cooking than a solid diet. Therefore, the volume is larger than that of a solid diet. After surgery, patients with OC do not have sufficient swallowing capacity to eat large amounts of food due to damaged or fatigued structures. Thus, BMI may be associated with days until removal of the NGT. The third explanation is that the relationship between the modified diet form and appetite might be related to days until removal of the NGT. As mentioned above, patients with dysphagia after surgery were forced to consume a modified diet due to the limited swallowing capacity. It has been reported that texture-modified diets are associated with poor appetite [30]. Thus, patients could not eat enough meals to remove the NGT due to this poor appetite. Given this background, we considered that a higher BMI was independently associated with the days until removal of the NGT. The nutritional status of patients with OC, including BMI before surgery, must be considered.

Regarding sex, removal of the NGT was more difficult for women than for men. It has been reported that dysphagia due to aging or sarcopenia was more common in men; this is in contrast to the results of the present study [31,32]. Given these facts, the attention we provide to patients with OC is different from that given to those with common dysphagia. Other variables that were not considered might have made it more difficult for women to undergo removal of the NGT. A previous study in patients with HNC reported that female patients presented significantly higher symptom scores of anxieties than male patients [33]. It has been reported that extensive oral cavity resection increases the risk of prolonged tube feeding requirements, which correlates with impaired aesthetic-related psychosocial functioning in patients with HNC [34]. Similarly, mental status may affect the desire to eat, which could indicate that it is more difficult for women to undergo removal of the NGT.

Our results showed a relationship between the presence of dysphagia before surgery and removal of the NGT. It has been reported that the preoperative FOIS scores are associated with the postoperative recovery of oral intake [8]. Thus, it is obvious that the presence of dysphagia before surgery accelerates the adverse effects of OC surgery on swallowing function.

In summary, the swallowing kinematics observed via VFSS were important for predicting removal of the NGT in patients with OC, as is the case in patients with common dysphagia. However, the influence of BMI was not similar to that seen in common dysphagia. This might be because the distribution of BMI, diet form after surgery, sufficient calories needed to be consumed, and appetite affected removal of the NGT. The findings according to sex were also not similar to those seen in common dysphagia. This might be because female patients with OC presented with significantly higher symptom scores for anxiety than male patients. Thus, to elucidate the association of these factors, further research is needed with greater participants and evaluation items for mental status, appetite, and calorie intake.

### 4.2. Other Nonsignificant Variables

Hyoid bone movement was not a significant variable affecting removal of the NGT in the log-rank test. Whether hyoid bone movement is correlated with dysphagia in patients with HNC remains controversial [12,35]. The results of this study could be because the suprahyoid muscles of patients with OC may or may not have been excised during surgery. Hyoid bone displacement originates from the contraction of the suprahyoid and infrahyoid muscles. The suprahyoid muscles play an important role in swallowing, but elevation of the hyolaryngeal complex during swallowing involves the supra- and infrahyoid muscles as well as other muscles, such as the pharyngeal muscle. Therefore, considering that UES opening was a predictive variable, assessing the escalation of thyroid cartilage, rather than hyoid elevation, might be more important in a clinical setting.

It is interesting to note that evaluation of swallowing kinematics, rather than aspiration, was a predictive variable. It has been reported that the presence of aspiration and/or penetration was associated with a lower probability of returning to complete oral feeding after stroke [36]. In addition, the same study found differences in cognitive scores between the resumption of oral feeding and the enteral nutrition groups. Furthermore, it has been reported that cognitive function might influence the recovery of oral intake ability [37]. In contrast, there were no patients with cognitive impairment after surgery in the present study. Thus, unlike in stroke patients, the absence of cognitive impairment might not be associated with removal of the NGT in patients with OC. The patients were instructed to learn compensatory strategies to avoid aspiration during meals. Compensatory strategies such as adequate diet form, posture during meals, and head rotation were determined according to VFSS [38]. All participants used compensatory strategies instructed by dentists for daily dysphagia rehabilitation. Thus, aspiration and pharyngeal residue were not significant variables for days until removal of the NGT.

Patients with OC have different prognoses from those with stroke or neuromuscular diseases, disuse by aging, accompanied by cognitive impairment, muscle atrophy, and disease progression. In addition, patients with OC treated with surgery or CR have damaged oropharyngeal structures or nerves that are not completely recovered. Thus, clinicians should assess oropharyngeal dynamics and residual swallowing function to ensure oral intake recovery after surgery.

### 4.3. Clinical Implications

In this study, swallowing kinematics was an important evaluation item when considering the prognosis of oral intake in patients with OC. Among the swallowing kinematics to be evaluated, UES and PCR_N_ should be evaluated by VFSS, whereas LVC should be evaluated by FEES in addition to VFSS. The finding that aspiration on the first VFSS after surgery was not a significant variable was considered to be because the compensatory strategies were effective in patients with OC. Additionally, it was difficult for patients with a large BMI to undergo removal of their NGTs because of the need to consume large amounts of calories through restricted diets. Female patients with OC exhibited similar findings because of their mental status. Clinicians might have to assess psychosocial function before and after surgery, and psychosocial intervention may be needed in some cases. Moreover, clinicians might have to recommend PEG for patients with several of these risk factors combined.

### 4.4. Limitations

This study has some limitations. First, we could not divide the participants into groups according to the type of cancer and operation performed due to the small numbers in the subgroups. It has been reported that surgery or CR for HNC affects swallowing kinematics, such as the oral preparatory, oral, and pharyngeal phases [2,3]. We could not adjust for these confounding factors. However, Table 2 shows that there was no difference in days until removal of the NGT according to the type of cancer. The type of cancer and surgical procedure might not be directly associated with removal of the NGT. Further studies are needed to consider these points. Second, appetite was not evaluated. Appetite is often affected by pain, discomfort, mental status, and preference in patients with HNC and is associated with oral intake. We could not collect data on appetite because this was a retrospective study in which a questionnaire that evaluated appetite or mental status related to appetite was not used. Finally, there was a selection bias among the participants. They were all selected for evaluation of swallowing function by oral surgeons at the same institution after surgery. As there was a variation in participant selection, those included in this study did not show similar cancer stages and exhibited a non-normally distributed age range.

## 5. Conclusions

The present study shows that PCR_N_, UES opening, LVC, sex, dysphagia, and BMI before surgery are risk factors for removal of the NGT in patients with OC. Clinicians should pay attention to these items, rather than aspiration, on the first VFSS because postoperative compensatory strategies are effective for patients with OC. Moreover, clinicians should assess the risk of aspiration or diet form for safe eating, predict how swallowing function recovers, and determine when to optimally remove the NGT.

## Figures and Tables

**Table 1:** Baseline characteristics of patients with oral (*n* = 129).

Variable		*n* (%)	
Sex	Male	66	(51.2)
	Female	63	(48.8)
Age	<65	54	(41.9)
	≥65	75	(58.1)
BMI	<18.5	14	(10.9)
	≥18.5	115	(89.1)
FOIS	1	7	(5.4)
	2	0	(0)
Stage	3	0	(0)
	4	10	(7.8)
	5	3	(2.3)
	6	18	(14.0)
	7	91	(70.5)
	I	10	(7.8)
	II	27	(20.9)
	III	35	(27.1)
	IV	57	(44.2)
Type of cancer	Tongue	61	(47.3)
	Gingival	44	(34.1)
	Upper jaw	11	(8.5)
	Lower jaw	33	(25.6)
	Buccal mucosal	10	(7.8)
	Oral floor	10	(7.8)
	Lower jaw	2	(1.6)
	Hard plate	1	(0.7)
	Myxoma	1	(0.7)

Abbreviations: BMI, body mass index; FOIS, functional oral intake scale.

**Table 2:** Days until removal of the nasogastric tube according to the type of cancer.

Type of Cancer		Days until Removal of the Nasogastric Tube	*n*	(%)	*p*-Value *
Tongue	median (IQR)	19 (16.0–25.0)	61	(47.3)	0.266
Gingival	median (IQR)	18.5 (14.3–24.5)	44	(34.1)	
Upper jaw	Mean ± SD	18.9 ± 12.0	11	(8.5)	
Lower jaw	median (IQR)	17.0 (15.0–24.0)	33	(25.6)	
Buccal mucosal	Mean ± SD	25.3 ± 11.8	10	(7.8)	
Oral floor	Mean ± SD	20.7 ± 7.5	10	(7.8)	
Lower jaw		23, 28	2	(1.6)	
Hard plate		30	1	(0.7)	
Myxoma		14	1	(0.7)	
Overall	IQR	20.5 (18.6–28.0)	129	(100.0)	

Abbreviations: IQR, interquartile range; SD, standard deviation *p*-value *: Kruskal–Wallis test adjusted with Bonferroni correction.

**Table 3:** Predictors of days until removal of the nasogastric tube, assessed using a Cox proportional hazards model.

Variable		Unadjusted HR	95%CI		*p*-Value	Adjusted HR	95%CI		*p*-Value
Sex	(female)	1.02	0.71	1.48	0.900	0.57	0.36	0.88	0.012 *
Age	(elderly)	0.73	0.49	1.08	0.118	0.87	0.57	1.32	0.510
BMI	(heavy)	0.80	0.44	1.43	0.443	0.42	0.22	0.84	<0.014 *
Dysphagia before surgery	(+)	0.4	0.23	0.73	<0.001 **	0.27	0.14	0.51	<0.001 **
CR before surgery	(+)	0.70	0.47	1.03	0.073	0.60	0.38	0.95	0.03 *
Aspiration	(+)	0.78	0.51	1.20	0.256	0.67	0.42	1.08	0.091
OPD	(−)	1.54	1.06	2.27	0.023 *	0.69	0.43	1.12	0.141
LVC	(bad)	2.04	1.39	3.03	<0.001 **	0.52	0.34	0.833	0.005 **
PCR_N_	(Large amount)	0.58	0.39	0.84	0.004 **	0.58	0.37	0.90	0.014 *
UES opening	(small amount)	0.52	0.36	0.77	<0.001 **	0.41	0.26	0.65	<0.001 **
Hyoid bone displacement	(Large amount)	0.74	0.51	1.08	0.118	0.69	0.41	1.15	0.157

Abbreviations: HR, hazard ratio; BMI, body mass index; CR, chemotherapy and/or radiotherapy; OPD, oral phase dysphagia; PCR_N_, pharyngeal constriction ratio normalized; UES opening, upper esophageal sphincter; LVC, laryngeal vestibule closure. *: *p* < 0.05, **: *p* < 0.01. Note: Table 3 shows how sex, BMI before surgery, dysphagia before surgery, CR, LVC, PCR_N_, and UES opening affected the period until removal of the NGT.
